# Perspectives of parents of adolescents with repeated non-suicidal self-injury on sharing their caretaking experiences with peers: a qualitative study

**DOI:** 10.3389/fpsyt.2023.1237436

**Published:** 2023-12-11

**Authors:** Yuan Qin, Dongmei Wu, Jiao Liu, Jianyan Peng, Chunya Li

**Affiliations:** ^1^School of Nursing, Chengdu Medical College, Chengdu, China; ^2^Chongqing Mental Health Center, Chongqing, China; ^3^Department of Nursing, The Clinical Hospital of Chengdu Brain Science Institute, MOE Key Laboratory for Neuroinformation, University of Electronic Science and Technology of China, Chengdu, China; ^4^Nursing Key Laboratory of Sichuan Province, Chengdu, China; ^5^School of Nursing, Zunyi Medical University, Zunyi, China

**Keywords:** non-suicidal self-injury, adolescents, parents, share, peers, qualitative study

## Abstract

**Background:**

The prevalence of non-suicidal self-injury among adolescents has increased over the years. Most parents, however, are poorly informed and confused about this behavior. Sharing caretaking experiences with parents in similar situations seems to be beneficial. Nevertheless, few researchers have explored the views of parents who share their caretaking experiences with peers.

**Aim:**

This study aimed to investigate the perspectives of parents of adolescents with repeated non-suicidal self-injury on sharing their caretaking experiences with peers as well as the motivations for and barriers to this behavior.

**Methods:**

This qualitative study adopted a purposive sampling method. Participants (16 mothers and 2 fathers) were recruited from the mental health center of a tertiary hospital in Chengdu, Sichuan, China. A total of 18 semistructured face-to-face individual interviews were conducted. All interviews were audio-recorded and analyzed thematically using NVivo 11.

**Results:**

Three themes and nine subthemes were identified: (1) sharable caretaking experiences: reflection and transformation, self-emotional management, and diversified support; (2) motivations for sharing: empathy, reciprocity, and meaning; and (3) barriers to sharing: inadequate knowledge, low self-identity, and concerns for children.

**Conclusion:**

Parents accumulate a wealth of experience during their long-term care of adolescents with repeated non-suicidal self-injury. Although most parents are willing to share their caretaking experiences with peers, there are several barriers. Therefore, in order to increase parents’ motivation to share, psychological education is necessary.

## Introduction

Non-suicidal self-injury (NSSI) refers to behaviors in which individuals directly and deliberately damage their body tissues with no intention of suicide (1), including actions such as cutting, biting, burning, hitting, and scratching (2, 3). Although the functions of self-injury remain unclear, a prior review reported various motivations for this behavior, including reducing distress, inflicting self-punishment and/or signaling personal distress to others (4). NSSI is particularly prevalent in adolescence (5). Some data suggest that the prevalence of NSSI has increased over the years (6), and a recent meta-analysis revealed that the global prevalence in a nonclinical sample of adolescents between 2010 and 2021 was 23.2% (7). In a sample of Chinese adolescents, the prevalence rate of at least one incident of NSSI was as high as 29% (8). Additionally, NSSI often occurs repeatedly (9). A case–control study on NSSI found that approximately 45.24% of cases reported recurrent NSSI (10). Consequently, these behaviors are related to higher rates of lifetime NSSI among adolescents (11). Moreover, the presence of NSSI is associated with a considerable risk of future suicidality (2, 12). A cross-sectional study of 600 adolescents found that more than half of adolescents with NSSI presented a significant suicide risk (13). In addition, findings from school-based studies indicate that high family conflict is a significant risk factor for NSSI and suicide (14).

NSSI is a transdiagnostic behavior that co-occurs with many mental diseases, including depressive disorder, anxiety disorder, borderline personality disorder, panic disorder, schizophrenia, and posttraumatic stress disorder (15, 16). Adolescent NSSI is strongly associated with complex factors such as social contagion, interpersonal stressors, neurobiological background, emotional dysregulation, and adverse experiences in childhood (5). There is also evidence that factors related to the family are associated with the occurrence and maintenance of adolescent NSSI (17), such as poor family functioning (18) and problematic parent–child relationships (19). Notably, a negative coping style among parents predicts increased odds of NSSI onset (20), which is closely related to repeated NSSI and severe NSSI in adolescents (21).

The impacts of young people’s self-injury on their families can be devastating. Many parents describe feelings of emotional distress, anger, shame, and blame (22). These reactions aggravate their children’s negative emotions and lead to serious consequences (23). According to a previous study, social isolation has been reported as parents withdraw from social contact due to the perceived stigma associated with NSSI (24). A qualitative study showed that parents of adolescents who engage in self-injury often experience significant distress and functional impairment, resulting in hypervigilance that adversely affects their mental health and social functioning (25). While social support (26) and professional support (27) are recommended for parents, there are numerous barriers (28). Previous studies have reported that stigma associated with mental illness, negative perceptions of healthcare services, a shortage of professionals, and long waiting times for professional assistance are common interpersonal barriers that prevent parents from seeking professional help (29, 30). These testimonies highlight the need for additional resources that better align with the needs of parents and that supplement professional assistance.

Peers are people with similar life experiences (31). Previous studies have shown that peers offer support outreach, engagement, knowledge, and care coordination to caregivers of adolescents with mental health problems (32, 33). Contact with peers also helps to establish trusting relationships between families, reduces the stigma of mental illness (34) and negative emotions (35), and improves coping mechanisms (36). According to a previous study, parents of adolescents with NSSI often feel ashamed to consult other parents with normal children. Instead, they tend to seek help from other parents in similar situations (37, 38). Sharing allows them to exchange caretaking experiences and improve their understanding of NSSI from different perspectives (38). Nonetheless, it is unclear how parents’ views are expressed through the sharing of caretaking experiences with peers.

Previous qualitative studies of parents of adolescents with NSSI have focused on experiences with and attitudes toward NSSI (22, 24, 38–40), with little attention to parents’ views on sharing caretaking experiences with peers. Therefore, this study aims to investigate the perspectives of parents of adolescents with repeated NSSI on sharing their caretaking experiences with peers as well as their motivations for and barriers to sharing. We hope to provide more accessible intellectual and emotional support resources to supplement professional assistance.

## Materials and methods

### Study design and setting

This descriptive, qualitative interview study was performed with parents of adolescents with repeated NSSI behaviors. The study was conducted from August 2022 to November 2022 at a 208-bed-capacity mental health center of a tertiary hospital in Chengdu, Sichuan, China. The center specializes in providing multidisciplinary mental health services for children and adolescents with various mental health problems.

### Research team

The research team consisted of two graduate students and three psychiatric nurses with 8 years, 12 years, and 20 years of experience in psychiatry. All researchers received qualitative research training before the interviews.

### Recruitment of participants

Purposive sampling was used to recruit the participants. The recruited participants were fathers and mothers of adolescents (age 13–18) with a history of at least two episodes of NSSI. The participants were required to have lived with the adolescents since the first self-injury. Informed consent was obtained from all participants for their own participation. The forms, frequency of NSSI, and parental mental state were determined by clinical data recorded by psychiatrists at the first consultation. Diversity in gender, age, residence, educational background, and marriage was considered in the sampling. Interviews were conducted until data saturation was attained. Eighteen participants completed interviews, including 16 mothers and 2 fathers.

### Data collection

Eighteen semistructured face-to-face individual interviews were conducted in private rooms in the hospital. The interviews were audio recorded and began with open-ended questions. Data were collected following a flexible thematic outline. The interview outline was developed based on previous literature and was revised after consultation with psychiatrists and psychiatric nurses. Subsequently, a pre-interview was conducted with two participants to evaluate the applicability of the outline. The outline included the following content: (1) What useful caretaking experiences have you had that can be shared with parents of adolescents with NSSI? (2) Would you like to share your caretaking experiences? (3) What factors motivate you to share your caretaking experiences? (4) What factors deter you from sharing your caretaking experiences? Depending on the participants’ preferences, the interviews were conducted in Mandarin or the Sichuan dialect. To ensure the privacy of the participants, they were referred to as P1 ~ P18. Each interview lasted 45 to 60 min.

### Data analysis

The audio recordings were transcribed into text and analyzed using NVivo 11. Braun and Clarke’s six-phase thematic analysis was conducted, which is an accessible and theoretically flexible approach to analyzing qualitative data and identifying themes or patterns (41). In the first phase, two researchers read the raw transcripts multiple times to familiarize themselves with the data and identify relevant ideas. In the second phase, these relevant ideas were reviewed several times and categorized according to their nature and frequency, and meaningful codes were developed from the transcript by two independent researchers. The reliability of coding was assessed by evaluating the degree of consensus. In case of disagreement on the codes, all researchers compared and discussed the coding to achieve consensus. In the third phase, related codes were grouped into categories and potential themes, which were labeled with the closest available approximation to the meaning. Two researchers reviewed different clustering conditions and searched for negative cases. The use of negative case analysis allows for the exploration and discussion of all data, including those that were inconsistent with the developed themes, which results in a more thorough understanding and fairness of the data (42). In the fourth phase, the codes, categories, and themes were compared between cases to ensure comprehensiveness and accuracy. The relationships between each theme were explored to generate a “thematic map” for the entire data set. In the fifth phase, each theme’s label was defined by an explanatory statement that unified its codes in terms of consistency and meaning. At several time points across the entire coding process, all researchers met to discuss, review, and triangulate the codes and themes to form the final codebook. The last phase involved writing the reports and ensuring their effectiveness for clinical implementation. To calculate interrater reliability, we used percentage agreement (#inter-rater agreements/total #extracts coded; mean = 96%; range = 92–100%). The final set of themes and subthemes was organized by all researchers, and disagreements were discussed until all members reached a consensus. When reporting the results, quotes from participants were used to explain the results. We have tried to approach the literal translation, and errors specific to the native language have been corrected to improve readability.

### Ethics

The study was ethically approved by the Ethics Committee of Chengdu Fourth Hospital on 24 March 2022: [2022] Lun Shen Zi No. (02), and the registration number of the Chinese Clinical Trial Registry was ChiCTR2200059437. Before each interview, written informed consent was obtained from all participants, and all of them were informed of the purpose, procedure and voluntary nature of the interview.

### Trustworthiness

Credibility, dependability, and transferability were considered to assess trustworthiness in this study (43). To achieve credibility, at the beginning of the interviews, the interviewer explained to the participants that their experiences were valuable and that they could express these experiences freely. Additionally, credibility was ensured through the diversity of participants, in-depth interviews with participants, and member checking by returning the transcripts to the participants for clarification. Dependability was established by external checks with advisory experts, including the transcripts, codes, categories, and themes, to identify any disagreement in the process of data collection and data analysis. In addition, we asked questions on the same subjects for all participants, and an observer observed the conduct of the interviews to record the participants’ non-verbal communication (e.g., facial expressions, tone, and mood). Transferability was achieved by purposeful sampling with maximum variation and a clear description of the data. In addition, all phases of the study were explained in detail to the participants. The participants’ statements were faithfully translated into English with intact preservation of the original statements.

## Results

**Figure 1:**
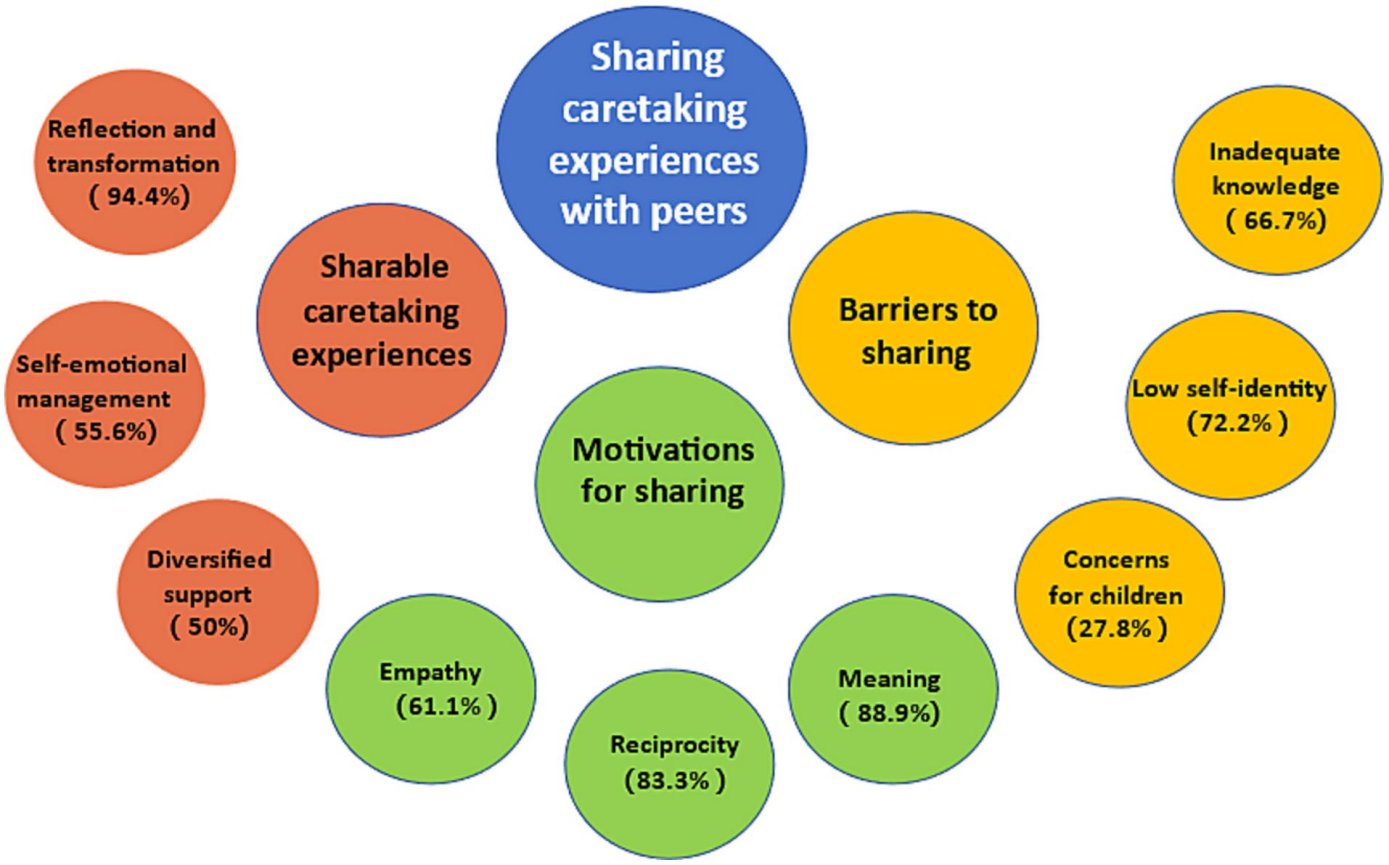
Thematic map for three major themes (middle circle) and nine subthemes (small circle).

Table 1 shows the demographic characteristics and relevant information on NSSI. Based on the eighteen interviews, three themes and nine subthemes were identified: (1) sharable caretaking experiences: reflection and transformation, self-emotional management, and diversified support; (2) motivations for sharing: empathy, reciprocity, and meaning; and (3) barriers to sharing: inadequate knowledge, low self-identity, and concerns for children. For each subtheme, we calculated the percentage of participants who mentioned it (Figure 1).

**Table 1:** Demographic characteristics and relevant information on NSSI.

		Adolescent			Participant				
No.	Age	Gender	NSSI method	NSSI frequency	Age	Gender	Residence	Education level	Marital status
1	15	Female	a	>5	44	Female	City	Bachelor degree	Married
2	14	Female	a	2–5	39	Female	City	Primary school	Married
3	15	Male	a	>5	39	Female	City	Bachelor degree	Married
4	16	Female	a, b	>5	51	Female	Countryside	Primary school	Remarried
5	15	Female	a, c	>5	44	Male	City	Primary school	Divorced
6	14	Female	a, c, d	2–5	55	Female	City	Bachelor degree	Married
7	16	Female	a	>5	51	Female	City	Some college	Married
8	14	Female	a, c, e	>5	45	Female	Countryside	Primary school	Remarried
9	13	Female	a	>5	34	Female	Countryside	Primary school	Remarried
10	14	Female	c, e	2–5	51	Female	Countryside	Middle school	Married
11	13	Female	a	2–5	51	Female	City	Some college	Married
12	17	Female	a, c, b	2–5	50	Female	Countryside	Middle school	Married
13	15	Male	a, c, d	2–5	49	Female	City	High school	Married
14	16	Female	a, b	>5	39	Male	City	High school	Married
15	14	Female	a, d	>5	45	Female	City	High school	Married
16	17	Female	a	>5	41	Female	City	Middle school	Married
17	18	Female	a	>5	47	Female	City	Middle school	Divorced
18	13	Male	a, d, f	2–5	34	Female	City	Middle school	Married

a, cutting; b, overdosing on medication; c, scratching; d, pinching; e, head banging; f, biting.

### Theme 1: sharable caretaking experiences

#### Subtheme 1: reflection and transformation

Some participants showed an understanding of NSSI in adolescents during long-term care. They pointed to the need for parents to reflect on the potential causes of NSSI from their perspective.

*“… These problems also prompted us to reflect on why our children injured themselves. The surface phenomenon of self-injury is not the most important, but the root cause..” (P13)*

*“… Self-injury should be taken seriously from the first time. I think we should take more care of our children in all aspects and promptly discover the state of our children in any situation …”(P15)*

Many participants emphasized the importance of parents learning about NSSI and psychology.

*“… I think we should learn something about NSSI, such as coping methods and ways to relieve children's negative emotions …” (P9)*

*“… I used to communicate with her rudely. After I learned the relevant psychological knowledge, I realized that some of my previous methods ignored my child's feelings, which reflected a lack of parenting skills, so I think parents must learn something about it..” (P12)*

*“… I think what we have to learn is how to communicate with adolescents and deal with NSSI …” (P2, P5, P15)*

#### Subtheme 2: self-emotional management

Most parents described negative emotions toward their adolescent who was self-injuring, including shock, anger, blame, and guilt. These reactions may worsen the situation. Therefore, some participants noted the need to manage their negative emotions.

*“… Many parents may blame themselves when learning about their children’s self-injury. But I want to advise parents not to blame themselves excessively in this situation because the more we blame, the harder it is for the child to get out.” (P3)*

*“… I used to lose my temper and lash out at my kids (crying), but I realized it was wrong. We have to control our emotions; only in this way can the children get better …” (P4)*

*“… At that time, my emotions affected her and made her feel guilty, so she hurt herself again. Only when parents are stronger and control their emotions can children feel better …” (P14)*

#### Subtheme 3: diversified support

Many participants commented that it was impossible to help children with NSSI by relying only on themselves; the combined efforts of the whole family were required.

*“… Indeed, the family atmosphere is very important; both parents and grandparents need to make progress. The whole family should support her …” (P6)*

Some participants explained that school had a great influence on children and that teachers’ attitudes were crucial. Parents should maintain close contact with teachers and seek their support.

*“… The influence of the school environment should not be ignored. I wanted to arouse the teachers' attention, so I exaggeratedly communicated with teachers …” (P2)*

*“… I talked to teachers and explained that my child was sick and may hurt himself when he got upset, so I hoped teachers would pay more attention to his emotions rather than academic performance. He needed his teachers’ support at this stage …” (P13)*

*“… I told the teacher about the situation of the child's self-injury and asked her not to put too much pressure on my child …” (P17)*

When parents initially learned that their children had NSSI behaviors, many of them were reluctant to admit that their children were ill and refused to take them to a psychiatrist. They expressed regret for this behavior and suggested that other parents seek timely professional help.

*“… It may have been two or three years since the first time my child hurt herself, but we did not notice. It was only when it got worse that we realized that we should take her to the hospital. So I suggest that other parents should bring their children to psychiatrists as soon as possible …” (P6)*

*“… If I had brought my child to the doctor when she began to hurt herself, it would not have been as serious as it is now. I advise that parents ought to seek medical help as soon as possible when they find something wrong with their children …” (P10)*

### Theme 2: motivations for sharing

#### Subtheme 1: empathy

Having experienced similar situations, many participants expressed empathy for the pain and helplessness of other parents of adolescents with NSSI.

*“… Since my child was hospitalized recently, I have found that too many children have self-injury behaviors. It breaks my heart (frowning), and I wonder what I would do if I were the mother of this child …” (P1)*

*“… Many parents were confused, and they did not know what to do, nor did I. I am willing to share my experience with other parents to prevent them from taking some detours …” (P2)*

*“… Some parents just need the help of parents with similar experiences; otherwise, they do not understand their children’s self-injury behaviors at all. It upsets me to see them in pain …” (P11)*

Some participants emphasized the importance of emotional communication, noting that sharing their caretaking experiences with peers could provide them with hope and emotional support.

*“… In a course I attended before, there was a session in which parents with similar life experiences shared some experiences of caring for adolescents with self-injury. I was deeply inspired, and I saw hope in them. I think that parents in similar situations can encourage each other in this way …” (P4)*

*“… Knowing the fact about my child's transition from severe self-injury to ceasing self-injury may increase their confidence …” (P16)*

#### Subtheme 2: reciprocity

Many participants expressed that sharing was a process of mutual learning that may encourage parents in similar situations to move forward together.

*“… I think I learned a lot every time I communicated with other parents. Because each child and each family are different, the approach adopted may be different, and I can learn useful strategies from them …” (P1)*

*“… In fact, this is a process of joint growth …” (P7, P17)*

Some participants admitted that they had significant deficits in dealing with their children’s NSSI behaviors, and they hoped to receive advice from other parents.

*“… I would like to share some changes in the way I get along with my child, and I am also glad that other parents can point out my deficiencies …” (P5)*

*“… Sharing my experiences in caring for my child with other parents, I think that other parents can apply what I did well, and I also hope that they provide some suggestions on my shortcomings …” (P14)*

Furthermore, given the stigma associated with mental illness, parents of adolescents with NSSI are commonly self-isolated. Some of the participants mentioned that sharing caretaking experiences with peers effectively reduced their level of isolation.

*“… Most parents are afraid to discuss this with parents of normal children, and they are frequently in isolation. Communication between parents of children exhibiting self-injury is necessary …” (P8)*

*“… I think the form of sharing is very useful. At least, It makes parents feel like they're not alone. …” (P13)*

#### Subtheme 3: meaning

Many participants found it meaningful and rewarding to help peers by sharing their caretaking experiences, and they strongly endorsed the process of sharing.

*“… If other parents benefit from my experience, it is a good thing …” (P8)*

*“… It is positive, right, and meaningful; how can I not do it?” (P10)*

*“… It would be great if I shared my experience with other parents and gave them some experience. It makes sense to help them …”(P15)*

Several participants considered this action fulfilling, as helping other parents may boost their sense of self-worth.

*“… I am willing to share because doing that is very meaningful. Although I am a full-time mother, I also want to realize my value …” (P2)*

Some participants expressed hope that more people would recognize NSSI behaviors in adolescents through sharing, thereby drawing society’s attention to the psychological problems of adolescents.

*“… I just think there should be a person with such consciousness to arouse more attention from society and make some improvements …” (P1)*

*“… Due to the lack of publicity and awareness of NSSI, most people have no understanding of these behaviors, including teachers. We want to share it so that more people recognize it and ultimately increase the government’s and public’s awareness of it …” (P8)*

### Theme 3: barriers to sharing

#### Subtheme 1: inadequate knowledge

Many participants reported that their low educational level was the main factor that impeded their willingness to share caretaking experiences with peers.

*“… I am not well educated, and I do not know what to share …” (P4, P12)*

*“… My low level of education results in me having nothing to share …” (P16)*

Other participants believed that their understanding of NSSI was not sufficient for them to share their experiences and expressed concerns that they would not be able to share useful knowledge.

*“… I think it is necessary to study systematically. Because what I learned may only scratch the surface, just some of my own experience …” (P4)*

*“… I do not think I have anything to share because I am just rich in theoretical knowledge, which has not been applied to practice …” (P13)*

*“… What I can share is my own experience, which is not professional. I am afraid I cannot say anything professional …” (P18)*

#### Subtheme 2: low self-identity

Many participants believed that they had failed after learning about their children’s NSSI behaviors, and they expressed a lack of confidence in sharing their caretaking experiences with peers.

*“… I think the main reason for my child’s self-injury was that I put too much pressure on her and did not care about her. So I think I am a failure as a parent …” (P2)*

*“… I feel like a failure, and I do not know what I can share with other parents …” (P7, P12)*

Some participants obtained relevant psychological knowledge but still felt helpless due to their children’s uncooperation.

*“… I have learned something about psychology, but now I have no clue because he is not willing to communicate with me (sighing). So I feel I have no experience to share …” (P3)*

*“… I have learned about nonviolent communication with my child, but I think it does not work. He is so resistant that he does not talk to me at all …” (P13)*

Most participants expressed concerns about sharing their caretaking experiences with peers due to their limited communication skills.

*“… The main reason is that I am not eloquent; if I am asked to share my experience with other parents formally, I think I may not be able to say anything …” (P8)*

*“… I think my oral expression ability is poor, I am afraid I cannot say anything …” (P9, P16)*

#### Subtheme 3: concerns for children

Some participants reported that their children’s attitudes had a strong influence on their willingness to share, and they worried that sharing their experiences may negatively impact their children.

*“… The main thing is that my child is very sensitive. I am afraid that she will be angry if she finds out I'm sharing with other parents. …” (P10)*

*“… She would not allow me to tell anyone about her condition, not even the doctor, let alone anyone else …” (P17)*

## Discussion

To the best of our knowledge, this study is the first to investigate the perspectives of parents of adolescents with repeated NSSI on sharing their caretaking experiences with peers. We identified three themes: sharable caretaking experiences, motivations for sharing, and barriers to sharing. During their long-term care for adolescents with repeated NSSI, many parents accumulated a wealth of experience that they believed could be shared with their peers. These topics included reflection and transformation, self-emotional management, and diversified support. Most of them expressed great willingness to share with their peers due to their empathy for the difficulties and helplessness that other parents faced, and they believed that sharing was reciprocal and meaningful. However, there were some barriers to sharing, such as inadequate knowledge, low self-identity, and concerns for children.

In agreement with a previous study (44), the present study revealed that many parents develop significant caretaking experiences during the long-term care of adolescents with NSSI. Most parents initially viewed NSSI as a manifestation of rebellion in the context of puberty (39). They also perceived NSSI as a way for children to threaten their parents or achieve certain goals (22). As the symptoms aggravate gradually, many parents began to reflect on themselves. They also mentioned the importance of making changes and continuous learning (39). In addition, some parents claimed that paying too much attention to this behavior may aggravate children’s guilt and blame (44). Parents also suggested to adjust their own negative emotions by participating in self-care activities, such as hobbies, mindfulness skills, spirituality techniques, and breathing exercises (38). Parents may benefit from diversified assistance, including formal resources and informal resources. On the one hand, parents may receive professional help to deal with the pressures and difficulties they face. On the other hand, parents may benefit from the support of friends, relatives, and peers to manage the impact of NSSI behaviors on their lives (37).

The majority of parents were willing to share their experiences of caring for adolescents with NSSI with peers. Considering the stigma related to mental disorders, they tended to be reluctant to seek psychological assistance for their child but sought a more general helping response from people close to them (22). Sharing has been reported to reduce the internalized stigma associated with mental illness and promote empowerment and hope (45) as well as a sense of belonging (46). In this study, many participants stated that sharing caretaking experiences with peers was meaningful because most parents struggle to understand or know how to react when their child self-injures (47). In previous studies, the importance of trust, respect, and empathy in this process was emphasized, and communication between parents facing similar challenges served as a source of information and emotional support (47, 48). Furthermore, sharing was considered reciprocal; for peer providers, sharing helped them obtain valuable advice from other parents (34). Specifically, sharing was beneficial for improving mental illness management and general health and enhancing parents’ emotional lives and self-awareness. Moreover, it helped parents reshape their sense of meaning, life perspective, and personal development as well as interpersonal relations (49).

Although most parents reported motivations and intentions to share their caring experiences with peers, there were several barriers. In agreement with Tang et al. (8) the majority of parents of adolescents with NSSI had low levels of education. Many parents stated that they had not heard of this behavior before their child hurt himself/herself for the first time. It was only after they realized the seriousness, they began to reflect and adapt (39). Consequently, they feared that they were not equipped with the abilities to appropriately support other parents. Consistent with Krysinska’s research (38), many parents had a low sense of self-identity, and they considered themselves failures. This lack of confidence is considered to be the main obstacle for them to share with peers (50). In addition, given that many adolescents with NSSI refuse to disclose these behaviors to others (51), many parents fear that sharing information about their children without their consent may increase family conflict and thus lead to severe NSSI in adolescents. Parents’ attitudes toward adolescents who self-injure can make a considerable difference in engagement and motivation (52). Given the evidence that psychoeducation can improve communication, increase knowledge, and change attitudes (47), providing parents with relevant psychoeducation may enhance their motivation to share.

Currently, psychotherapy is considered the main treatment for NSSI (53). However, a prior survey indicated that approximately 56.2% of adolescents with NSSI have not received formal psychiatric therapy (54). Additionally, there is a shortage of psychotherapists in China, and the distribution of psychotherapists is uneven (55). Therefore, making supplementary resources available to parents is essential. The interviews with parents of adolescents who repeatedly engaged in NSSI allowed us to summarize the key points of the caretaking experiences, which may equip concerned parents with appropriate knowledge by sharing. Furthermore, parents’ motivations and barriers to sharing were explored. This study provides a basis for parents of adolescents with NSSI to seek additional resources and emotional support.

## Limitations

Despite the promising results of this study, there are some limitations. First, our findings can only be generalized to a certain extent since the participants consisted of 16 mothers and 2 fathers, and their perceptions might reflect parents’ views rather than the perspectives of a wider range of relatives. Second, because most adolescents were accompanied by their mothers while hospitalized, the majority of participants were mothers. Therefore, our findings might be more representative of mothers’ views, which may create significant biases in the research results and generalizability. Third, all participants were parents of hospitalized patients. Interviewing parents of adolescents with NSSI who have not received any professional assistance would provide a more comprehensive understanding of parents’ sharing of caretaking experiences with their peers. Future research should compensate for the shortcomings mentioned above.

## Conclusion

The current study highlights the perspectives of parents of adolescents with repeated NSSI on sharing their caretaking experiences with peers as well as their motivations and barriers. In view of the general lack of NSSI knowledge among parents, psychoeducation is necessary to overcome barriers to sharing. It is suggested that professionals recruit experienced parents as volunteers to teach them theoretical knowledge and practical skills in the future. In this way, these parents will be capable of providing additional intellectual and emotional support to complement professional assistance, thereby increasing parents’ access to assistance.

## Data availability statement

The raw data supporting the conclusions of this article will be made available by the authors, without undue reservation.

## Ethics statement

The studies involving humans were approved by the Ethics Committee of Chengdu Fourth Hospital. The studies were conducted in accordance with the local legislation and institutional requirements. The participants provided their written informed consent to participate in this study. Written informed consent was obtained from the individual(s) for the publication of any potentially identifiable images or data included in this article.

## Author contributions

DW was responsible for study design and quality control section. YQ and JL were responsible for data collection and analysis. JP and CL were responsible for recruiting and screening participants and revised the interview outline. YQ drafted the manuscript, DW revised the manuscript. All authors reviewed the manuscript and gave final approval of the version to be published.
